# A novel electronic health record-based, machine-learning model to predict severe hypoglycemia leading to hospitalizations in older adults with diabetes: A territory-wide cohort and modeling study

**DOI:** 10.1371/journal.pmed.1004369

**Published:** 2024-04-12

**Authors:** Mai Shi, Aimin Yang, Eric S. H. Lau, Andrea O. Y. Luk, Ronald C. W. Ma, Alice P. S. Kong, Raymond S. M. Wong, Jones C. M. Chan, Juliana C. N. Chan, Elaine Chow

**Affiliations:** 1 Department of Medicine and Therapeutics, The Chinese University of Hong Kong, Prince of Wales Hospital, Hong Kong, China; 2 Li Ka Shing Institute of Health Sciences, The Chinese University of Hong Kong, Prince of Wales Hospital, Hong Kong, China; 3 Hong Kong Institute of Diabetes and Obesity, The Chinese University of Hong Kong, Prince of Wales Hospital, Hong Kong, China; 4 Phase 1 Clinical Trial Centre, The Chinese University of Hong Kong, Prince of Wales Hospital, Hong Kong, China

## Abstract

**Background:**

Older adults with diabetes are at high risk of severe hypoglycemia (SH). Many machine-learning (ML) models predict short-term hypoglycemia are not specific for older adults and show poor precision-recall. We aimed to develop a multidimensional, electronic health record (EHR)-based ML model to predict one-year risk of SH requiring hospitalization in older adults with diabetes.

**Methods and findings:**

We adopted a case-control design for a retrospective territory-wide cohort of 1,456,618 records from 364,863 unique older adults (age ≥65 years) with diabetes and at least 1 Hong Kong Hospital Authority attendance from 2013 to 2018. We used 258 predictors including demographics, admissions, diagnoses, medications, and routine laboratory tests in a one-year period to predict SH events requiring hospitalization in the following 12 months. The cohort was randomly split into training, testing, and internal validation sets in a 7:2:1 ratio. Six ML algorithms were evaluated including logistic-regression, random forest, gradient boost machine, deep neural network (DNN), XGBoost, and Rulefit. We tested our model in a temporal validation cohort in the Hong Kong Diabetes Register with predictors defined in 2018 and outcome events defined in 2019. Predictive performance was assessed using area under the receiver operating characteristic curve (AUROC), area under the precision-recall curve (AUPRC) statistics, and positive predictive value (PPV). We identified 11,128 SH events requiring hospitalization during the observation periods. The XGBoost model yielded the best performance (AUROC = 0.978 [95% CI 0.972 to 0.984]; AUPRC = 0.670 [95% CI 0.652 to 0.688]; PPV = 0.721 [95% CI 0.703 to 0.739]). This was superior to an 11-variable conventional logistic-regression model comprised of age, sex, history of SH, hypertension, blood glucose, kidney function measurements, and use of oral glucose-lowering drugs (GLDs) (AUROC = 0.906; AUPRC = 0.085; PPV = 0.468). Top impactful predictors included non-use of lipid-regulating drugs, in-patient admission, urgent emergency triage, insulin use, and history of SH. External validation in the HKDR cohort yielded AUROC of 0.856 [95% CI 0.838 to 0.873]. Main limitations of this study included limited transportability of the model and lack of geographically independent validation.

**Conclusions:**

Our novel-ML model demonstrated good discrimination and high precision in predicting one-year risk of SH requiring hospitalization. This may be integrated into EHR decision support systems for preemptive intervention in older adults at highest risk.

## Abstract 2

Using nearly 1.5 million health records from >350,000 older adults with diabetes in Hong Kong, Elaine Chow and colleagues investigate a novel machine learning model to predict risk of severe hypoglycaemia.

## Author summary

**Why was this study done?:**

- Older adults with diabetes are at high risk of severe hypoglycemia (SH) requiring hospitalization.
- Existing machine-learning (ML) models predict short-term hypoglycemia are not specific for older adults and show poor precision-recall.
- A simple tool to identify those at risk for developing SH in T2D is needed.

**What did the researchers do and find?:**

- We included 1,456,618 records of 364,863 unique older adults (age ≥65 years) with diabetes and at least 1 Hong Kong Hospital Authority attendance in 2013 to 2018.
- We used 258 predictors including demographics, admissions, diagnoses, medications, and routine laboratory tests in a one-year period to predict SH events requiring hospitalization in the following 12 months.
- Six ML algorithms were evaluated including logistic-regression, random forest, gradient boost machine, deep neural network (DNN), XGBoost, and Rulefit.
- The XGBoost model yielded the best performance, superior to an 11-variable conventional logistic-regression model.

**What do these findings mean?:**

- Our novel-ML model demonstrated good discrimination and high precision in predicting one-year risk of SH requiring hospitalization.
- This may be integrated into electronic health record (EHR) decision support systems for preemptive intervention in older adults at highest risk.
- A limitation of this study is the lack of model validation in independent cohorts outside Hong Kong.

## Introduction

Severe hypoglycemia (SH), different from general hypoglycemia by the requirement of assistance from a third party, is a feared complication in the management of diabetes in older adults [1]. According to the multicenter *Hypoglycemia Assessment Tool (HAT) Study*, 83% of people with type 1 diabetes (T1D) and 46.5% of insulin-treated people with type 2 diabetes (T2D) had ever reported hypoglycemia [2,3]. Multiple risk factors contribute to increased risk of SH in older adults including long disease duration, decline in hypoglycemia awareness, renal impairment, cognitive dysfunction, and insulin use [3]. In Hong Kong, people with diabetes aged ≥75 years had the highest rate of hospitalization due to SH compared with younger adults aged 45 to 59 years (6.0 versus 2.9 events/100-person-years) [4]. Apart from prolonged hospitalization and high healthcare expenditure, SH is associated with increased risk of cardiovascular (CV) disease, falls, dementia, and all-cause mortality [5]. In a recent survey, most US physicians rarely de-intensified or switched hypoglycemia-causing medications in high-risk older adults [6]. International guidelines recommend screening for “geriatric syndromes” including polypharmacy as part of an extended diabetes complication assessment in older adults [7]. This calls for a systematic paradigm for predicting SH risk in older adults, followed by personalized prevention and treatment strategies to avoid SH events and related comorbidities [8]. There is a need for a model specifically designed for SH in older adults with diabetes, as compared to risk prediction in the general population of people with diabetes.

SH risk prediction models have traditionally been developed using physiological and clinical variables, utilizing conventional statistical methods [9–13]. Karter and colleagues proposed a 6-variable risk stratification tool that categorized patients’ 12-month risk of hypoglycemia-related emergency department (ED) attendance or hospitalization [9]. The predictors included number of episodes of hypoglycemia-related utilization, insulin use, sulfonylurea (SU) use, prior year emergency room use, kidney disease, and age (c-statistic of 0.83). Majority of these models demonstrated high performance in terms of area under the receiver operating characteristic curve (AUROC) reaching over 80% [9–13]. However, since SH is relatively rare in people with diabetes, the high AUROC of a prediction model may be driven by the accurate distinguishment of those at extremely low risk of SH (i.e., true negatives), who were usually the majority in the training cohorts. In such unbalanced datasets, it may be more important to maximize precision-recall, or the ability to predict the rare occurrence of a positive SH event [14]. A high proportion of false positives could lead to unnecessary intervention or de-intensification of treatment in low-risk individuals and inefficient resource utilization. Unfortunately, few published electronic health record (EHR)-based models for SH have evaluated precision-recall. In a recent study, Ruan and colleagues used an EHR-based model with laboratory and clinical variables to predict short-term inpatient hypoglycemia [15]. The best performing model was based on a machine-learning (ML) algorithm XGBoost which yielded both high AUROC and precision-recall.

Time series records of clinical variables are necessary for developing models that forecast SH events [16]. Hong Kong has a unique territory-wide EHR system that covers 90% of older adults aged 65 or above in the city [17]. In this study, making use of the comprehensive, multidimensional data available in the local EHR system, we aimed to develop a novel ML-based model for predicting one-year risk of SH requiring hospitalization in older adults with diabetes. We anticipated that the proposed model could be embedded in a decision support system (DSS) to provide regular SH risk screening for older Chinese people with diabetes.

## Materials and methods

### Dataset

In Hong Kong, the Hospital Authority (HA) established a Big Data Analytics Platform, namely Hospital Authority Data Collaboration Lab (HADCL), to support and facilitate territory-wide secure sharing of EHR-based dataset. HADCL provides anonymized data covering a broad range of patient information collected from all public hospitals and clinics in Hong Kong. The EHR system has provided an integrated, longitudinal, lifelong view of patient’s health status and clinical outcomes, including comprehensive medication and laboratory records, hospitalization, residential area (linked to poverty index), health service utilization, comorbidity, and procedure data [17]. We extracted a retrospective dataset from the HADCL, consisting of patients aged 65 years or above with any in-patient admissions or out-patient attendances from January 1, 2013 to December 31, 2018. All data used was collected for routine patient management with no additional data input required for the modeling [18]. The dataset contains patient demographics, living districts, utilization of health care resources (in-patient admissions, transfer and discharge, out-patient admissions, ED attendance), disease diagnosis based on the International Classification of Diseases Ninth Revision (ICD-9) codes, medication dispensing data, and laboratory tests. Personal information was removed during the analysis procedure. Ethics approval was obtained from the Joint Chinese University of Hong Kong-New Territories East Cluster Clinical Research Ethics Committee (CREC-2021.050).

### Study reporting

This study is reported as per transparent reporting of a multivariable prediction model for individual prognosis or diagnosis (TRIPOD) guideline (**S1 Checklist**).

### Study population

People with diabetes were defined by those meeting any one of the following criteria [4]: (1) a diagnosis code for diabetes based on ICD-9 code of 250.xx from specialist out-patient clinics (SOPCs) and during hospitalization; (2) diagnosis code for diabetes based on the International Classification of Primary Care, Second Edition (ICPC-2): T89 or T90 at the general out-patient clinics (GOPCs); (3) HbA1c ≥6.5% in any 1 available measurement; (4) fasting plasma glucose (FPG) ≥7.0 mmol/L in any 1 available laboratory measurement; (5) prescription of any glucose-lowering drugs (GLDs); or (6) long-term prescription of insulin for at least 28 consecutive days.

### Study design and outcome

The current study adopted a case-control design. The primary outcome for the cases was hospitalization due to SH, as defined by the principal hospital discharge diagnosis ICD-9 codes (250.80, 250.81, 250.82, 250.83, and 250.30–250.33) [4]. A detailed description of the definition was summarized in **S1 Table**.

### Predictors

We curated multidimensional variables from the integrated EHR dataset [1,19], which included sociodemographic characteristics, living districts (which are linked to the average income of the residents as an index of social deprivation and poverty), utilization of health care resources (including admissions, clinic visits, consultations by allied healthcare professionals, emergency room visits), disease diagnosis from ICD-9 codes, medication dispensing data, and laboratory data (hematology, renal and liver function tests, glycemic and lipid indexes). These variables were selected based on published literature, prior knowledge, and data availability within EHR. We curated a total of 258 predictors for model derivation. A full list of the predictors and how they were represented in the source data systems and in the prediction models is available in **S2 Table**.

### Prediction horizon and observational period

The prediction horizon (PH) is the time period between model forecasting and the occurrence of a predicted event [20]. In this study, we adopted a PH of 12-month as a balance of SH event rate and clinical utility. To enrich the number of events available for model training, we allowed individuals to have multiple SH events during the whole investigation period.

**Fig 1:**
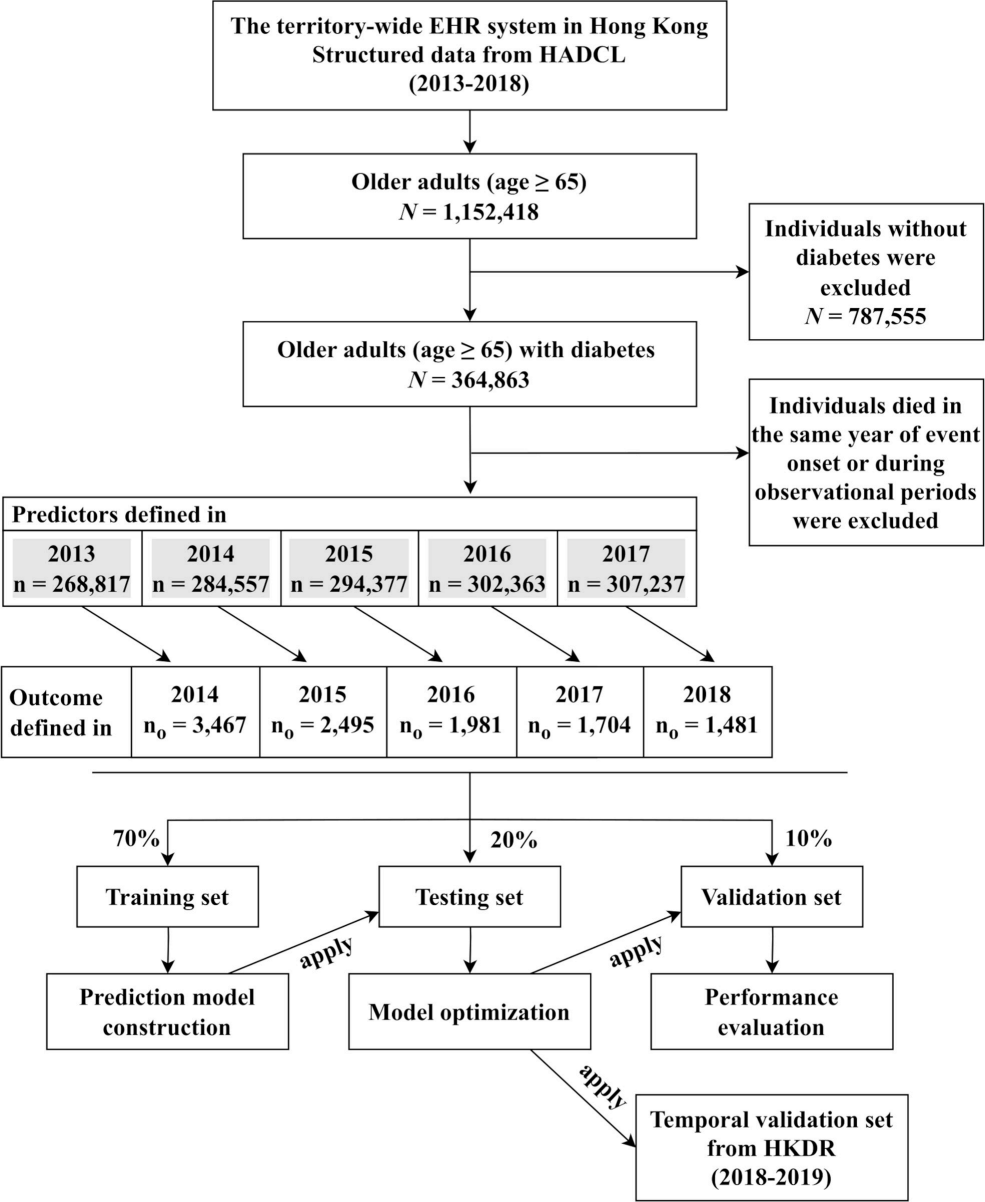
Study Design. *N*, number of individuals; n, number of records; n_o_, number of outcome events.

We defined a 12-month period prior to the date of hospitalization due to SH as the observational period of the cases. For subjects free of SH events, we used the calendar year as the observational periods in controls. We excluded individuals who died in the same year of event onset for cases and during the observational period for controls. We calculated summary statistics for laboratory predictors within the observational periods (mean, median, maximum, and minimum) which are referred to as annual-based values hereinafter. We used 5 consecutive years (2013 to 2017) of data to develop a model that predicted SH events leading to hospitalizations in the subsequent 12 months (2014 to 2018) (**Fig 1**).

### Missing data

Considering the missingness in EHR data is not at complete random, we additionally defined dummy variables for laboratory predictors as the surrogates of the factors that led to the missingness. We discarded the annual-based values of predictors with missingness >50% and only retained the dummy indicators as the surrogates of these predictors [21]. We imputed the missing values using the cohort median for the remaining features.

### Model development

We applied 6 supervised ML algorithms for training the SH risk prediction models [22]. These algorithms included generalized linear model (GLM), distributed random forest (DRF), gradient boosting machine (GBM), Rulefit, deep neural network (DNN), and extreme gradient boosting (XGBoost). The whole cohort was randomly split into training (70%), testing (20%), and validation sets (10%) (**Fig 1**). The models were developed using the training set and optimized via hyper-parameter tuning in the testing set. For benchmarking, we also applied the same 6 ML algorithms to train the models but using only 11 variables that were previously reported to predict hypoglycemia. The best 11-variable model approximates a conventional strategy of SH risk prediction (9–13). These risk factors included age, sex, history of SH, hypertension, blood glucose (HbA1c and FPG), urinary albumin-to-creatinine ratio, estimated-glomerular filtration rate (eGFR) derived from serum creatinine using the Chronic Kidney Disease Epidemiology Collaboration (CKD-EPI) equation, use of metformin, SU, and insulin. We evaluated the models derived from different algorithms and hyper-parameters based on their performance in the validation set. The model development was conducted on the H2O platform (package version: 3.36.1.1) in R environment (www.r-project.org) [23].

### Hyper-parameter optimization

When training the ML models, we conducted hyper-parameter tuning using either the default setting or a random grid search strategy implemented in the H2O package. In particular, we specified a set of values for the key hyper-parameters that affected learning rate of each ML algorithm (**S3 Table**). By random grid search, all possible combinations of hyper-parameter values were sampled uniformly from the hyper-parameter space to train the model. We then selected the combination of hyper-parameters that optimized the area under the precision-recall curve (AUPRC) in the testing data for the final models.

### Model comparison

We evaluated the model performance using discrimination and calibration metrics. Given the limitations in sole considering AUROC as a discrimination metric, we also considered precision and recall. The former, also known as positive predictive value (PPV), is a measure of the ability of the model to correctly predict a patient as having hypoglycemia, computed by true positive/(true positive + false positive). The latter, known as sensitivity, is a measure of the ability of the model to label as hypoglycemic all of patients who did indeed develop hypoglycemia, signified by the ratio true positives/(true positive + false negatives). The AUPRC was computed at the threshold that yielded the maximum *F1* score in the validation set. The *F1* score, calculated by the harmonic mean of the precision and recall, measures how well the prediction model can correctly identify all the positive cases and meanwhile avoid making mistakes by marking a negative control as positive. We considered the model with the highest AUPRC as the best. Calibration, the extent to which the predicted risk scores accurately estimate the observed values, was visually assessed by a calibration plot. We compared the observed and predicted risk of SH at 12-month in the validation set by ranking subjects into deciles of predicted risk. In addition, we generated risk probabilities for the outcome event using the best ML model in the training data, and scaled the probabilities to align with a continuous score from 1 to 100 by uniform quantile transformation. We then applied this scaling scheme to the validation set. Score cut-off that enriched 90% of events in the validation data was selected as the threshold for risk stratification.

### External temporal validation

To assess the performance of the developed model [24], we performed validation in a separate temporal cohort selected from the Hong Kong Diabetes Register (HKDR) [18]. The HKDR is an ongoing prospective register-based cohort of individuals with diabetes since 1995 who have undergone structured diabetes assessments at one of the HA hospitals, Prince of Wales Hospital, Hong Kong SAR. The HKDR cohort was periodically linked to the territory-wide Clinical Management System (CMS) for capturing of laboratory data, treatment, hospitalizations, and death. Characteristics of patients in the HKDR are described elsewhere [18]. This validation cohort was composed of patients with diabetes aged 65 years or above in 2018 and alive by the end of 2019. The same definitions for predictors, outcome, and observational periods were used as in the previous analysis.

### Variable importance

We sought to understand how the different variables contributed to the predictions by the XGBoost model (the selected best predictive model). We calculated the variable importance using tree-based algorithms by the H2O platform. The variable importance is computed from the gains of their respective loss functions during tree construction [25]. Additionally, we used Shapley additive explanation (SHAP) value to understand the contribution of each predictor variable in the temporal validation cohort [26].

### Sensitivity analysis

To interrogate the transferability of our ML model, we additionally performed 2 sensitivity analyses by restricting the predictors to the top 30 variables revealed by variable importance of our XGBoost model. We first re-trained the model using all the 30 predictors using the same training dataset. We then re-trained the second model using 22 predictors that were selected from the top 30 and were considered to be more accessible in routine healthcare and less region-specific. These excluded predictors were mainly outpatient specialty, triage category during ED attendance, ward care type and length of stay during inpatient admission, procedure times, and district of residence. The re-trained models were optimized in the testing cohort and then evaluated in both internal and external temporal validation cohorts.

### Statistical methods

We presented descriptive statistics as means (standard deviations) or medians (interquartile ranges) to characterize individuals across different years or groups. The ANOVA and χ^2^ tests were employed to compare differences across multiple groups for continuous and categorical variables, respectively.

## Results

### Demographic characteristics

From January 1, 2013 to December 31, 2017, we identified 1,456,618 patient records of 364,863 individuals with diabetes aged above 65 with valid observational periods (2013 to 2017). The mean age was 74.4 ± 8.0 years and 46.6% of the patients were male. A total of 9,616 unique patients had been hospitalized due to SH during PHs, from which we identified 11,128 outcome events. The prevalence of hospitalization due to SH had declined from 1.3% in 2014 to 0.4% in 2018 (**Table 1**). Compared with patients without SH hospitalizations, patients who developed SH hospitalizations were older (77.9 ± 7.6 versus 74.4 ± 8.0 years), had more in-patient (3.7 versus 2.1 times/years), out-patient records (18.2 versus 15.1 times/year), and had history of SH (10.0% versus 0.7%). Meanwhile, they were more likely to be taking SU (67.4% versus 43.4%), insulin (48.2% versus 12.3%), and dipeptidyl peptidase-4 inhibitors (DPP-4is) (16.9% versus 7.8%), but were less likely to be taking lipid-regulating drugs (14.9% versus 67.7%) (**S4 Table**). Distributions of baseline characteristics were similar in training, testing, and validation sets (**S4 Table**).

**Table 1:** Characteristics of SH requiring hospitalization among old adults with diabetes in 2013–2017.

Characteristics	2013	2014	2015	2016	2017	*P* value
Number of patients	268,817	284,557	294,377	302,363	307,237	<0.001
Outcome events in 12 months (%)	3,467 (1.3)	2,495 (0.8)	1,981 (0.6)	1,704 (0.5)	1,481 (0.4)	<0.001
Male (%)	124,166 (46.2)	132,199 (46.5)	137,189 (46.6)	141,196 (46.7)	143,555 (46.8)	<0.001
Age; mean (SD)	73.2 (8.2)	73.8 (8.1)	74.4 (8.0)	75.0 (7.8)	75.6 (7.7)	<0.001
Age group (%)						<0.001
65–69	106,441 (39.6)	105,557 (37.1)	102,568 (34.9)	96,008 (31.8)	85,216 (27.8)	
70–79	96,253 (35.8)	103,837 (36.5)	108,964 (37.0)	116,409 (38.5)	125,256 (40.8)	
80–89	58,978 (21.9)	66,051 (23.2)	71,809 (24.4)	77,105 (25.5)	81,726 (26.6)	
90+	7,194 (2.7)	9,023 (3.2)	10,861 (3.7)	12,624 (4.2)	14,736 (4.8)	
In-patient records; mean (SD)	2.0 (8.9)	2.1 (9.3)	2.1 (9.1)	2.2 (9.1)	2.3 (9.4)	<0.001
In-patient records; median [IQR]	0.0 [0.0, 2.0]	0.0 [0.0, 2.0]	0.0 [0.0, 2.0]	0.0 [0.0, 2.0]	0.0 [0.0, 2.0]	<0.001
Out-patient records; mean (SD)	14.9 (15.8)	14.8 (16.1)	15.1 (16.1)	15.3 (16.2)	15.5 (16.5)	0.024
Out-patient records; median [IQR]	11.0 [7.0, 18.0]	11.0 [7.0, 17.0]	11.0 [7.0, 18.0]	11.0 [7.0, 18.0]	11.0 [7.0, 19.0]	<0.001
Glucose-lowering drugs (%)						
Metformin	185,541 (69.0)	187,983 (66.1)	189,424 (64.4)	191,128 (63.3)	191,329 (62.3)	<0.001
Sulfonylurea	134,296 (49.9)	130,812 (46.0)	126,730 (43.1)	123,242 (40.8)	119,261 (38.9)	<0.001
DPP4-i	13,936 (5.2)	17,638 (6.2)	22,538 (7.7)	27,438 (9.1)	32,518 (10.6)	<0.001
TZD	1,100 (0.4)	1,202 (0.4)	1,755 (0.6)	4,200 (1.4)	6,754 (2.2)	<0.001
GLP1-RA	53 (<0.1)	69 (<0.1)	87 (<0.1)	99 (<0.1)	134 (<0.1)	<0.001
SGLT2i	0	0	253 (0.1)	1,360 (0.5)	2,928 (1.0)	<0.001
Insulin	33,786 (12.6)	34,939 (12.3)	36,573 (12.4)	37,666 (12.5)	39,398 (12.8)	<0.001
Lipid-regulating drugs (%)	164,534 (61.2)	185,058 (65.1)	200,444 (68.1)	212,892 (70.5)	221,071 (72.0)	<0.001
History of insulin use (%)	35,120 (13.1)	36,535 (12.8)	38,645 (13.1)	40,268 (13.3)	42,597 (13.9)	<0.001
History of hospitalized SH (%)	3,091 (1.1)	2,737 (1.0)	1,977 (0.7)	1,622 (0.5)	1,404 (0.5)	<0.001

SD, standard deviation; IQR, interquartile range; DPP4-i, dipeptidyl peptidase-4 inhibitors; TZD, thiazolidinediones; GLP1-RA, glucagon-like peptide-1 receptor agonists; SGLT2i, sodium-glucose cotransporter 2 inhibitors; SH, severe hypoglycemia.

### Model performance

All the ML algorithms, including the conventional models using only 11 variables, yielded high AUROC value above 0.8 in training, testing, and validation sets (**Tables 2** and **S5**). Among them, the model based on XGBoost algorithm had the best performance concerning false positives and false negatives in the internal validation set (AUPRC = 0.670; PPV = 0.848). The best ML model based on 11 conventional variables, however, only yielded an AUPRC of 0.280 (XGBoost algorithm; **S5 Table**). We assessed the model calibration by splitting the validation set into deciles ordered by predicted probability of risk, where the XGBoost-based model demonstrated a good concordance between the observed and predicted events (**S1 Fig**). We selected a scaled risk probability of 86 as the threshold for risk stratification since approximately 90% of cases were enriched in individuals with scaled scores greater than this cut-off in the validation dataset (**S6 Table**).

**Table 2:** Performance metrics of the ML models.

Machine-learning models	AUROC	AUPRC	PPV	NPV	F1	MCC
**Training set**						
XGBoost	0.989	0.795	0.852	0.998	0.747	0.751
Deep Learning	0.957	0.594	0.818	0.997	0.657	0.668
Gradient Boost Machine	0.828	0.542	0.576	0.995	0.413	0.428
Random Forest	0.932	0.375	0.634	0.995	0.378	0.411
RuleFit	0.932	0.406	0.579	0.995	0.426	0.439
Generalized Linear Model	0.884	0.091	0.521	0.995	0.344	0.363
**Testing set**						
XGBoost	0.977	0.694	0.819	0.997	0.671	0.681
Deep Learning	0.948	0.532	0.849	0.996	0.593	0.620
Gradient Boost Machine	0.933	0.462	0.521	0.995	0.356	0.372
Random Forest	0.929	0.455	0.637	0.995	0.419	0.443
RuleFit	0.904	0.393	0.576	0.995	0.415	0.429
Generalized Linear Model	0.878	0.097	0.520	0.995	0.338	0.358
**Validation set**						
XGBoost	0.978	0.670	0.848	0.997	0.642	0.660
Deep Learning	0.947	0.546	0.800	0.996	0.603	0.620
Gradient Boost Machine	0.936	0.470	0.445	0.995	0.354	0.358
Random Forest	0.927	0.467	0.798	0.995	0.422	0.476
RuleFit	0.911	0.380	0.580	0.995	0.405	0.422
Generalized Linear Model	0.881	0.096	0.345	0.995	0.345	0.354
**Temporal validation set**						
XGBoost	0.856	0.286	0.323	0.967	0.359	0.322

AUROC, area under the receiver operating characteristic curve; AUPRC, area under the precision-recall curve; PPV, positive predictive value (i.e., precision); NPV, negative predictive value; F1, F1 score calculated from the harmonic mean of the precision and recall; MCC, Matthews Correlation Coefficient; ML, machine-learning.

### Variable importance

**Fig 2:**
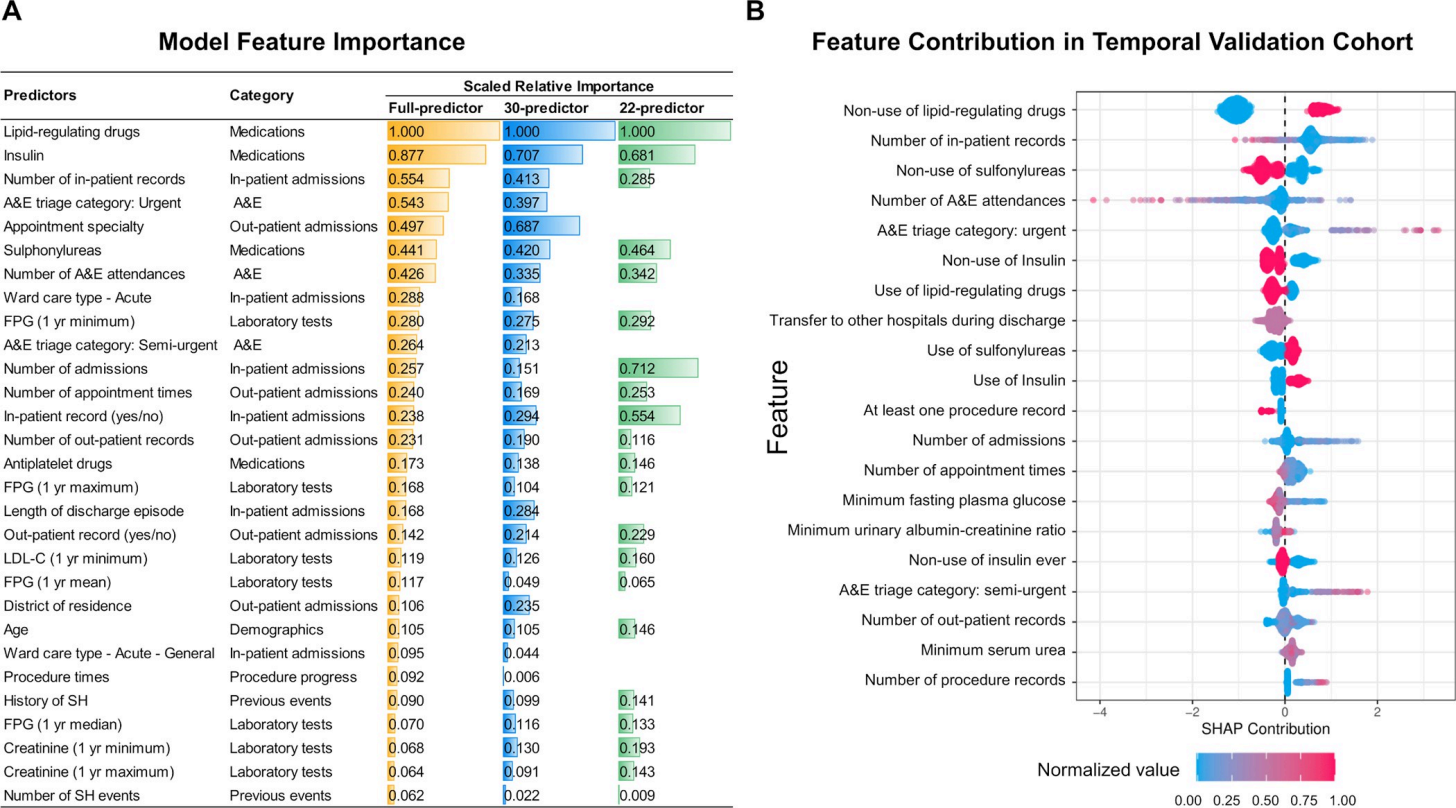
Scaled relative importance of top 30 predictors from the XGBoost model in the validation set (A) and contribution of the top 20 features of the XGBoost model in the temporal validation set (B). **(A)** Variable importance plot that shows the relative importance of top 30 predictors from the XGBoost model in the validation set. A&E, Accident and Emergency attendance; FPG, fasting plasma glucose; 1 yr minimum/mean/maximum, the minimum/mean/maximum of all the values of the corresponding laboratory test in the recent 12 months; LDL-C, low-density lipoprotein cholesterol. “Insulin” included both insulin use and ever use. **(B)** SHAP (SHapley Additive exPlanations) summary plot that shows the contribution of the top 20 features of the XGBoost model in the temporal validation set. Each feature corresponds to a continuous variable or a certain category of a categorical variable. One dot per subject per feature is colored according to the attribution value of the feature, where red represents a higher value (or “1” for a binary feature) and blue represents a lower value (or “0” for a binary feature). The features are ordered in a descending contribution to the XGBoost model. For example, non-use of lipid-regulating drugs (red color) is associated with the highest discriminative value for increased risk of SH (SHAP contribution >0), meanwhile, non-use of sulfonylureas (red color) associated with the discriminative value for reduced risk of SH (SHAP contribution <0).

**Fig 2A** demonstrates the top predictors out of 258 variables and their relative importance in the XGBoost model. The non-use of lipid-regulating drugs, use or historical use of insulin, number of in-patient records, triage category of “urgent” during ED attendance, and use of SU were the top 5 most important variables in the prediction model. Apart from well-established predictors such as medications, age, FPG, and history of SH, the XGBoost model also identified novel variables that could inform the risk of SH including, for example, outpatient appointment specialty, types of wards care, and the district of residence. Sensitivity analysis restricted to the top 30 predictors revealed that the predictive power of our model could be mostly retained by these top predictors (validation AUPRC = 0.632 versus 0.670 for the full-predictor model). Additional sensitivity analysis by further exclusion of region-specific predictors resulted in a 22-variable model with a moderate drop of performance (validation AUPRC = 0.540; **S5 Table**).

### External validation

To evaluate the robustness of the XGBoost-based prediction model against the training data collection period (2013 to 2018), we applied the final model to a temporal validation cohort from the HKDR. The HKDR cohort included predictors collected in 2018 and the occurrence of SH hospitalizations in 2019 and included 14,295 valid patients records in 13,917 patients aged 65 or above in 2018, diagnosed with diabetes, and alive by the end of 2019 (**Table 1**). Using the same outcome definition, we identified 722 SH hospitalizations. The XGBoost-based prediction model yielded an AUROC of 0.856 and an AUPRC of 0.286 in this separate cohort.

Top features revealed by the SHAP value showed high consistency, where non-use of lipid-regulating drugs in the recent 12 months had the largest discriminative power to indicate risk of hospitalizations due to SH. Fewer in-patient records, use of SU and insulin, more out-patient records, more urgent or semi-urgent triage at ED attendance, and lower annual-minimum of FPG were associated with increased risk of SH in subsequent 12 months in this separate validation cohort (**Fig 2B**).

## Discussion

SH poses a great healthcare burden for patients with diabetes, with potential life-threatening consequences particularly in older adults. In this study, we integrated comprehensive EHR and advanced ML algorithms to develop a risk prediction model for one-year SH hospitalization in older patients with diabetes. Compared with model built upon conventional predictors and algorithms, our model achieved improved AUROC and better precision-recall (AUPRC of 0.670 versus 0.097 for 11-variable generalized linear model). Based on routinely captured EHR data, this model has the potential to serve as a decision support tool that can be readily integrated into the territory-wide EHR system locally.

Although many models for hypoglycemia prediction have been proposed [15,27–31], the accuracy of these methods were only valid for short-term prediction in in-patient settings [22]. However, early prediction, which leaves the clinicians with sufficient time to adjust or redesign personalized therapeutic strategies, is more desirable for preventing SH in older adults. In addition, these models were prone to making false alarming, leading to inappropriate treatment deintensification and potentially increasing the risk of hyperglycemia. Our model achieved a good precision-recall of 0.670 given the prevalence of SH requiring hospitalization was only around 1%. In another ML model which was developed to predict near-term hypoglycemia, PPV was 0.09 [28].

Against this background, our EHR-based ML model offers a highly efficient and low-cost approach in predicting risk of hospitalization due to SH in 12 months in older adults with diabetes. Our model utilized annual-based summary statistics to reduce the variance and increase reliability of predictors. Our model relied on EHR data which can be updated in a real-time manner as the value of any included predictor changes. Our model was proposed in line with the aim of precision medicine, where more intensive monitoring and interventions for reducing risk of SH are focused on the minority of older patients in the high-risk category. In the majority of patients, the usual strategy to optimize glucose control can be adopted accompanied by education to increase the awareness of hypoglycemia. Given the close associations of age with many risk factors for SH, a model developed in an older age group will improve the precision in identifying the very high-risk subjects for corrective action without compromising the glycemic control in low-risk elderly patients.

In our ML model, we included more than 250 variables that were potentially predictive of SH hospitalizations. Our model also considered demographic variables like default for appointment specialty clinics and district of residence of higher index of deprivation as top predictors [32]. These associations reiterated the close inter-relationship among multiple morbidities including SH, fragility, and low socioeconomic status, which had not been highlighted by previous models [33,34]. Apart from confirming known clinical risk factors, such as use of insulin and SU, and history of SH events [3], our model has also revealed novel factors associated with SH. For example, non-use of lipid-regulating drug was identified as the most discriminative predictor of higher risk of SH in both the development and replication datasets. Although our analyses cannot be used to infer causation, the associations are plausible as statins are known to increase insulin resistance and worsen glucose tolerance [35]. Alternatively, non-use of lipid-lowering drug may be a marker of frailty or other shared risk factors for SH.

Our work has several strengths. This is the first risk prediction model for SH leading to hospitalizations in older adults with diabetes. We included over 1 million subjects for model training and validation, using over 250 multidimensional variables from a territory-wide EHR to build the model. We used annual-based summary statistics of variables to increase the stability of our model, making it less prone to errors due to outliers, sporadic data, or noisy laboratory test values which are common features in EHR data. We also benchmarked multiple supervised ML algorithms to obtain the optimized model. In addition to AUROC, we also presented AUPRC model that was often omitted by previous studies due to the rareness of SH events in previous database. Our advanced ML algorithms considered both nonlinear associations and interactions among predictors to identify both conventional and novel risk factors with better performance than conventional methods. The complexity of the model also takes into account the missing values of predictors, making it a useful decision support tool in a healthcare system. Finally, we validated our model using a temporal cohort that confirmed the robustness of our model for future prospective validation and implementation.

Our study also has limitations. First, we demonstrated temporal but not geographical transportability of our model. We utilized territory-wide dataset in Hong Kong across all public hospitals that are linked in our training dataset. The transportability of our model to other regions, countries, ethnicities, and healthcare systems is unknown given population characteristics are likely to be different. Similarly, the threshold we currently selected for risk stratification required recalibration when applying to other cohorts. However, as many of our top predictors and variables such as hospital attendance, drug use, and history of SH are commonly available in most EHR systems, we expect our work can inspire similar studies where our model can be adapted and calibrated to other settings. This was also supported by our sensitivity analyses where the model performance was still comparable when restricted to top 30 predictors. Second, our EHR system did not capture lifestyle-related variables (e.g., diet, exercise) or self-monitoring of blood glucose. Meanwhile, our data did not include anthropometric parameters either, which are important for dose-related calculations for medication exposure and can also reflect nutritional or health states. Finally, we used principal hospital discharge diagnostic codes to define SH events in this study, which may underestimate the number of SH events requiring third-party assistance but did not require hospital admission. Our model also does not predict non-SH which is mostly self-reported and not captured within EHRs. However, Karter and colleagues demonstrated their tool predicting 12-month hypoglycemia-related ED or hospital use showed high agreement with self-reported SH [9]. Further they demonstrated their tool also predicted continuous glucose monitoring (CGM) detected hypoglycemia (time <50 mg/dL) with high accuracy [36]. We plan to evaluate our ML model for non-severe and CGM-detected hypoglycemia in prospective studies.

In summary, we have developed a one-year EHR-based ML-risk prediction model for SH leading to hospitalizations in older adults with diabetes using multidimensional EHR data. The model outperforms conventional models in AUROC and precision-recall with reduced number of false positives which might lead to unnecessary interventions, with both implications for the patients and healthcare system. Given the increasing use of EHR, our ML model can be developed into a decision-making tool to alert physicians to implement early preventive actions, such as de-prescribing or treatment reconciliation. There is also growing evidence for use of technologies in older adults, such as CGM with hypoglycemic alerts [37,38]. We anticipate that the proposed model could be embedded in a DSS to provide regular SH risk screening for older Chinese people with diabetes [37]. Such program can be particularly effective if combined with a regular comprehensive diabetes assessment program that allows periodic review of clinical state for quality assurance [18]. Implementation studies are needed to define the logistics of ML-based hypoglycemia risk stratification tool with patient-centered decision support and evaluate its impacts on clinician and patient behavior, change of medications as well as clinical outcomes and cost-effectiveness.

## Supporting information

S1 TRIPOD Checklist Prediction model development and validation. (DOCX)

S1 Fig Observed and predicted number of severe hypoglycemia (SH) events in the validation set, by decile of predicted risk from the XGBoost-based, full-predictor model. (DOCX)

S1 Table Definition and description of hospitalized severe hypoglycemia in the electronic health record (EHR) system. (DOCX)

S2 Table List of candidate predictors available in the Hospital Authority Data Collaboration Lab (HADCL) cohort. (DOCX)

S3 Table Hyper-parameter values for machine-learning (ML) models. (DOCX)

S4 Table Cohort characteristics by outcome and dataset. (DOCX)

S5 Table Performance metrics of sensitivity analyses and 11-variable models. (DOCX)

S6 Table Scaled score for risk stratification in the validation set. (DOCX)

## Abbreviations

AUPRC: area under the precision-recall curve
AUROC: area under the receiver operating characteristic curve
CGM: continuous glucose monitoring
CV: cardiovascular
DNN: deep neural network
DRF: distributed random forest
DSS: decision support system
ED: emergency department
eGFR: estimated-glomerular filtration rate
EHR: electronic health record
FPG: fasting plasma glucose
GBM: gradient boosting machine
GLD: glucose-lowering drug
GLM: generalized linear model
GOPC: general out-patient clinic
HA: Hospital Authority
HADCL: Hospital Authority Data Collaboration Lab
HKDR: Hong Kong Diabetes Register
ML: machine-learning
PH: prediction horizon
PPV: positive predictive value
SH: severe hypoglycemia
SOPC: specialist out-patient clinic
T1D: type 1 diabetes
T2D: type 2 diabetes
