# Supplementary material for: A novel electronic health record-based, machine-learning model to predict severe hypoglycemia leading to hospitalizations in older adults with diabetes: A territory-wide cohort and modeling study
Source: PLoS Med. 2024 Apr 12;21(4):e1004369. doi: 10.1371/journal.pmed.1004369 (PMC11014435; doi:10.1371/journal.pmed.1004369)
Supplement: S1 Fig — (DOCX) [file pmed.1004369.s002.docx]

### S1 Fig. Observed and predicted number of severe hypoglycemia (SH) events in the validation set, by decile of predicted risk from the XGBoost-based, full-predictor model.
