# Supplementary material for: A novel electronic health record-based, machine-learning model to predict severe hypoglycemia leading to hospitalizations in older adults with diabetes: A territory-wide cohort and modeling study
Source: PLoS Med. 2024 Apr 12;21(4):e1004369. doi: 10.1371/journal.pmed.1004369 (PMC11014435; doi:10.1371/journal.pmed.1004369)
Supplement: S1 Table — (DOCX) [file pmed.1004369.s003.docx]

### S1 Table. Definition and description of hospitalized severe hypoglycemia in the electronic-health-record (EHR) system.

| **ICD‐9 codes** | **Full description in the EHR system** |
| --- | --- |
| 250.30 | Type II diabetes mellitus with hypoglycemic coma |
|  | Diabetes mellitus with hypoglycemic coma |
| 250.31 | Type I diabetes mellitus with hypoglycemic coma |
| 250.32 | Type II diabetes mellitus, uncontrolled with hypoglycemic coma |
|  | Diabetes mellitus, uncontrolled with hypoglycemic coma |
| 250.33 | Type I diabetes mellitus, uncontrolled with hypoglycemic coma |
| 250.80 | Type II diabetes mellitus with hypoglycemia |
|  | Type II diabetes mellitus with hypoglycemia, drug induced |
|  | Diabetes mellitus with hypoglycemia |
|  | Diabetes mellitus with drug induced hypoglycemia |
| 250.81 | Type I diabetes mellitus with drug induced hypoglycemia |
|  | Type I diabetes mellitus with hypoglycemia |
| 250.82 | Type II diabetes mellitus, uncontrolled with hypoglycemia |
|  | Type II diabetes mellitus, uncontrolled with drug induced hypoglycemia |
|  | Diabetes mellitus, uncontrolled with hypoglycemia |
|  | Diabetes mellitus, uncontrolled with drug induced hypoglycemia |
| 250.83 | Type I diabetes mellitus, uncontrolled with hypoglycemia |
|  | Type I diabetes mellitus, uncontrolled with drug induced hypoglycemia |

ICD-9: International Classification of Diseases Ninth Revision.
