# Supplementary material for: A novel electronic health record-based, machine-learning model to predict severe hypoglycemia leading to hospitalizations in older adults with diabetes: A territory-wide cohort and modeling study
Source: PLoS Med. 2024 Apr 12;21(4):e1004369. doi: 10.1371/journal.pmed.1004369 (PMC11014435; doi:10.1371/journal.pmed.1004369)
Supplement: S2 Table — (DOCX) [file pmed.1004369.s004.docx]

### S2 Table. List of candidate predictors available in the Hospital Authority Data Collaboration Lab (HADCL) cohort.

| **Predictor description** | **Data in model** | **Code in model** | **Remark/Unit** | **Range** | **Proportion of missing** |
| --- | --- | --- | --- | --- | --- |
| **Demographics** | | | | | |
| Age | continuous variable | *age* | years | 65 – 113 | <0.01% |
| Age groups | categorical:  65-69 years  70-79 years  80-89 years  90+ years | *age_group* |  | 4 categories | 0 |
| Sex | binary variable (0 = female, 1 = male) | *sex* |  | 0, 1 | 0 |
| **Out-patient admissions** | | | | | |
| Out-patient appointment | binary variable (yes, no) | *opas* |  | 0, 1 | 0 |
| Number of out-patient records | annual count, integer | *opas_records* |  | 0 – 1076 | 0 |
| Number of appointment times | annual count, integer | *appo_times* |  | 0 – 36 | 0 |
| Hospital Authority-wide standardized specialty | categorical variable at first appointment in each year | *appo_specialty* | Note 1* | 37 categories | 0.15% |
| District of residence | categorical variable: 19 districts based on hierarchical administrative subdivision codes | *appo_district* |  | 19 categories | 0 |
| **Accident and Emergency attendance (A&E)** | | | | | |
| Number of attendances | annual count, integer | *ae_times* |  | 0 – 270 | 0 |
| District of A&E admissions | categorical variable: 19 districts based on hierarchical administrative subdivision codes | *ae_district* |  | 19 categories | 65.90% |
| Number of triage category:  triage category: Critical  triage category: Emergent  triage category: Urgent  triage category: Semi-urgent  triage category: Non-urgent | annual count, integer | *ae_noTriage_1* |  | 0 – 9 | 0 |
|  |  | *ae_noTriage_2* |  | 0 – 25 | 0 |
|  |  | *ae_noTriage_3* |  | 0 – 77 | 0 |
|  |  | *ae_noTriage_4* |  | 0 – 223 | 0 |
|  |  | *ae_noTriage_5* |  | 0 – 56 | 0 |
| **Inpatient admissions, transfer and discharge** | | | | | |
| In-patient appointment | binary variable (yes, no) | *ipas* |  | 0, 1 | 0 |
| Number of in-patient records | annual count, integer | *ipas_records* |  | 0 – 1506 | 0 |
| District of admissions | categorical variable: 19 districts based on hierarchical administrative subdivision codes | *adm_district* |  | 19 categories | 0.29% |
| Number of admissions | annual count, integer | *adm_times* |  | 0 – 241 | 0 |
| Length of stay of discharge episode | annual length of stay for all admissions | *adm_length* | days | 0 – 1303 | 0 |
| Number of ward care type:  ward care type - Acute  ward care type - Conv/Reh  ward care type - Infirmary  ward care type - Others  ward care type - Acute - General  ward care type - Acute - Intensive Care Unit  ward care type - Acute - High Dependency Unit | annual count, integer | *adm_noWC_AG* |  | 0 – 241 | 0 |
|  |  | *adm_noWC_CONV* |  | 0 – 22 | 0 |
|  |  | *adm_noWC_INF* |  | 0 – 11 | 0 |
|  |  | *adm_noWC_OTH* |  | 0 – 15 | 0 |
|  |  | *adm_noWsub_GEN* |  | 0 – 241 | 0 |
|  |  | *adm_noWsub_ICU* |  | 0 – 3 | 0 |
|  |  | *adm_noWsub_HDU* |  | 0 – 5 | 0 |
| Type of case during discharge (first time) | categorical:  0 = missing  1 = transfer to other HA hospitals  2 = discharged home  3 = death  4 = other | *adm_destination* |  | 0, 1, 2, 3, 4 | 0 |
| **Diabetes Mellitus Complication Screening (DMCS)** | | | | | |
| DMCS indication | binary (yes, no) | *ramp_ever* |  | 0, 1 | 0 |
| **Procedure progress** |  |  |  |  |  |
| procedure indication | binary (0 = no procedure, 1 = at least one procedure) | *procedure_yes* |  | 0, 1 | 0 |
| Procedure times | annual count, integer | *procedure_times* |  | 0 – 160 | 0 |
| **Disease diagnosis** | | | | | |
| History of hospitalized severe hypoglycemia | binary (yes, no) | *sh* |  | 0, 1 | 0 |
| Number of hospitalized severe hypoglycemia | annual count, integer | *sh_n* |  | 0 – 7 | 0 |
| History of hospitalized fall | binary (yes, no) | *fall* |  | 0, 1 | 0 |
| Number of hospitalized fall | annual count, integer | *fall_n* |  | 0 – 9 | 0 |
| Cancer (exclude benign neoplasm, carcinoma in situ) | binary (yes, no) | *comp_e1* |  | 0, 1 | 0 |
| Dementia | binary (yes, no) | *comp_e2* |  | 0, 1 | 0 |
| Alcohol drug mental disorder | binary (yes, no) | *comp_e3* |  | 0, 1 | 0 |
| Transient psychotic disorder | binary (yes, no) | *comp_e4* |  | 0, 1 | 0 |
| Schizophrenia and psychoses | binary (yes, no) | *comp_e5* |  | 0, 1 | 0 |
| Other psych conditions | binary (yes, no) | *comp_e6* |  | 0, 1 | 0 |
| Other Cerebral degeneration | binary (yes, no) | *comp_e7* |  | 0, 1 | 0 |
| Parkinson’s disease & other move disorder | binary (yes, no) | *comp_e8* |  | 0, 1 | 0 |
| Spinal cord, autonomic | binary (yes, no) | *comp_e9* |  | 0, 1 | 0 |
| Neuropathy | binary (yes, no) | *comp_e10* |  | 0, 1 | 0 |
| Myoneural myopathy | binary (yes, no) | *comp_e11* |  | 0, 1 | 0 |
| Disorder of globe | binary (yes, no) | *comp_e12* |  | 0, 1 | 0 |
| Retinal and choroidal | binary (yes, no) | *comp_e13* |  | 0, 1 | 0 |
| Glaucoma | binary (yes, no) | *comp_e14* |  | 0, 1 | 0 |
| Cataract | binary (yes, no) | *comp_e15* |  | 0, 1 | 0 |
| Refraction | binary (yes, no) | *comp_e16* |  | 0, 1 | 0 |
| Visual disturbance | binary (yes, no) | *comp_e17* |  | 0, 1 | 0 |
| Blindness low vision | binary (yes, no) | *comp_e18* |  | 0, 1 | 0 |
| Diseases of arteries, arterioles, and capillaries | binary (yes, no) | *comp_e19* |  | 0, 1 | 0 |
| Keratitis, corneal, orbit | binary (yes, no) | *comp_e20* |  | 0, 1 | 0 |
| Optic nerve disease | binary (yes, no) | *comp_e21* |  | 0, 1 | 0 |
| Rheumatic valve | binary (yes, no) | *comp_e22* |  | 0, 1 | 0 |
| Hypertensive | binary (yes, no) | *comp_e23* |  | 0, 1 | 0 |
| Acute myocardial infarction (AMI) | binary (yes, no) | *comp_e24* |  | 0, 1 | 0 |
| Ischemic heart disease (IHD) | binary (yes, no) | *comp_e25* |  | 0, 1 | 0 |
| Pulmonary circ | binary (yes, no) | *comp_e26* |  | 0, 1 | 0 |
| Pericardial | binary (yes, no) | *comp_e27* |  | 0, 1 | 0 |
| Non rheumatic valve | binary (yes, no) | *comp_e28* |  | 0, 1 | 0 |
| Cardiomyopathy | binary (yes, no) | *comp_e29* |  | 0, 1 | 0 |
| Conduction and cardiac dysrhythmia | binary (yes, no) | *comp_e30* |  | 0, 1 | 0 |
| Heart failure | binary (yes, no) | *comp_e31* |  | 0, 1 | 0 |
| Intracerebral hemorrhage (ICH) | binary (yes, no) | *comp_e32* |  | 0, 1 | 0 |
| Cerebral ischemia/occlusion | binary (yes, no) | *comp_e33* |  | 0, 1 | 0 |
| Atherosclerosis, peripheral vascular disease (PVD),  embolic and thrombosis | binary (yes, no) | *comp_e34* |  | 0, 1 | 0 |
| Aneurysm | binary (yes, no) | *comp_e35* |  | 0, 1 | 0 |
| Phlebitis | binary (yes, no) | *comp_e36* |  | 0, 1 | 0 |
| Venous thrombosis | binary (yes, no) | *comp_e37* |  | 0, 1 | 0 |
| Gastric varices | binary (yes, no) | *comp_e38* |  | 0, 1 | 0 |
| Lymphatic disorder | binary (yes, no) | *comp_e39* |  | 0, 1 | 0 |
| Hypotension | binary (yes, no) | *comp_e40* |  | 0, 1 | 0 |
| Chronic obstructive pulmonary disease (COPD) | binary (yes, no) | *comp_e41* |  | 0, 1 | 0 |
| Asthma | binary (yes, no) | *comp_e42* |  | 0, 1 | 0 |
| Bronchiectasis | binary (yes, no) | *comp_e43* |  | 0, 1 | 0 |
| End-stage kidney disease (ESKD) | binary (yes, no) | *comp_e44* |  | 0, 1 | 0 |
| Chronic kidney disease (CKD) | binary (yes, no) | *comp_e45* |  | 0, 1 | 0 |
| Connective tissue diseases (CTD) & rheumatoid arthritis (RA) | binary (yes, no) | *comp_e46* |  | 0, 1 | 0 |
| Arthropathy infections | binary (yes, no) | *comp_e47* |  | 0, 1 | 0 |
| Osteoarthrosis and allied disorders | binary (yes, no) | *comp_e49* |  | 0, 1 | 0 |
| **Laboratory tests** | | | | | |
| Bilirubin in Serum or Plasma | annual average value | *lab_BIL_mean* | umol/L | 0.0 - 568.4 | 41.56% |
|  | annual median value | *lab_BIL_median* |  | 0.0 - 599.0 | 41.56% |
|  | annual maximum value | *lab_BIL_max* |  | 0.0 - 862.5 | 41.56% |
|  | annual minimum value | *lab_BIL_min* |  | 0.0 - 398.0 | 41.56% |
|  | availability, binary (yes, no) | *C_lab_BIL* |  | 0, 1 | 0 |
| High-density lipoprotein cholesterol (HDL-C) in Serum or Plasma | annual average value | *lab_HDLC_mean* | mmol/L | 0.1 - 6.2 | 24.28% |
|  | annual median value | *lab_HDLC_median* |  | 0.1 - 6.2 | 24.28% |
|  | annual maximum value | *lab_HDLC_max* |  | 0.1 - 6.2 | 24.28% |
|  | annual minimum value | *lab_HDLC_min* |  | 0.0 - 6.2 | 24.28% |
|  | availability, binary (yes, no) | *C_lab_HDLC* |  | 0, 1 | 0 |
| Total cholesterol (TC) in Serum or Plasma | annual average value | *lab_TC_mean* | mmol/L | 0.6 – 4.7 | 24.11% |
|  | annual median value | *lab_TC_median* |  | 0.6 – 4.7 | 24.11% |
|  | annual maximum value | *lab_TC_max* |  | 0.6 – 4.9 | 24.11% |
|  | annual minimum value | *lab_TC_min* |  | 0.6 – 4.6 | 24.11% |
|  | availability, binary (yes, no) | *C_lab_TC* |  | 0, 1 | 0 |
| Creatinine in Serum or Plasma | annual average value | *lab_Crea_mean* | umol/L | 14.33 - 1585.25 | 19.07% |
|  | annual median value | *lab_Crea_median* |  | 13.50 - 1597.00 | 19.07% |
|  | annual maximum value | *lab_Crea_max* |  | 18.0 - 2865.0 | 19.07% |
|  | annual minimum value | *lab_Crea_min* |  | 0.00 - 1484.00 | 19.07% |
|  | availability, binary (yes, no) | *C_lab_Crea* |  | 0, 1 | 0 |
| Glucose in Serum or Plasma | annual average value | *lab_FPG_all_mean* | mmol/L | 0.71 - 69.11 | 18.47% |
|  | annual median value | *lab_FPG_all_median* |  | 0.71 - 69.90 | 18.47% |
|  | annual maximum value | *lab_FPG_all_max* |  | 0.71 - 194.00 | 18.47% |
|  | annual minimum value | *lab_FPG_all_min* |  | 0.16 - 69.11 | 18.47% |
|  | availability, binary (yes, no) | *C_lab_FPG_all* |  | 0, 1 | 0 |
| Triglyceride (TG) in Serum or Plasma | annual average value | *lab_TG_mean* | mmol/L | 0.0 - 61.2 | 24.18% |
|  | annual median value | *lab_TG_median* |  | 0.0 - 61.2 | 24.18% |
|  | annual maximum value | *lab_TG_max* |  | 0.0 – 69.0 | 24.18% |
|  | annual minimum value | *lab_TG_min* |  | 0.0 – 57.1 | 24.18% |
|  | availability, binary (yes, no) | *C_lab_TG* |  | 0, 1 | 0 |
| Alanine aminotransferase (ALT) in Serum or Plasma | annual average value | *lab_ALT_mean* | U/L | 0.0 - 2391.2 | 37.87% |
|  | annual median value | *lab_ALT_median* |  | 0.0 - 2991.0 | 37.87% |
|  | annual maximum value | *lab_ALT_max* |  | 0.0 - 9165.4 | 37.87% |
|  | annual minimum value | *lab_ALT_min* |  | 0.0 - 601.9 | 37.87% |
|  | availability, binary (yes, no) | *C_lab_ALT* |  | 0, 1 | 0 |
| Albumin (Alb) in Serum or Plasma | annual average value | *lab_Alb_mean* | g/L | 10.1 - 55.1 | 41.45% |
|  | annual median value | *lab_Alb_median* |  | 10.0 - 55.1 | 41.45% |
|  | annual maximum value | *lab_Alb_max* |  | 13.8 - 68.0 | 41.45% |
|  | annual minimum value | *lab_Alb_min* |  | 2.0 - 55.1 | 41.45% |
|  | availability, binary (yes, no) | *C_lab_Alb* |  | 0, 1 | 0 |
| Urea in Serum or Plasma | annual average value | *lab_Urea_mean* | mmol/L | 0.60 - 69.33 | 19.29% |
|  | annual median value | *lab_Urea_median* |  | 0.60 - 69.95 | 19.29% |
|  | annual maximum value | *lab_Urea_max* |  | 0.60 - 114.60 | 19.29% |
|  | annual minimum value | *lab_Urea_min* |  | 0.00 - 54.11 | 19.29% |
|  | availability, binary (yes, no) | *C_lab_Urea* |  | 0, 1 | 0 |
| Potassium in Serum or Plasma | annual average value | *lab_K_serum_mean* | mmol/L | 2.07 - 9.51 | 19.31% |
|  | annual median value | *lab_K_serum_median* |  | 2.07 - 9.51 | 19.31% |
|  | annual maximum value | *lab_K_serum_max* |  | 2.07 - 16.00 | 19.31% |
|  | annual minimum value | *lab_K_serum_min* |  | 1.00 - 7.90 | 19.31% |
|  | availability, binary (yes, no) | *C_lab_K_serum* |  | 0, 1 | 0 |
| Protein in Serum or Plasma | annual average value | *lab_Protein_mean* | g/L | 35.0 - 127.5 | 41.56% |
|  | annual median value | *lab_Protein_median* |  | 34.0 - 128.7 | 41.56% |
|  | annual maximum value | *lab_Protein_max* |  | 39.0 - 149.0 | 41.56% |
|  | annual minimum value | *lab_Protein_min* |  | 12.0 - 125.0 | 41.56% |
|  | availability, binary (yes, no) | *C_lab_Protein* |  | 0, 1 | 0 |
| Sodium in Serum or Plasma | annual average value | *lab_Na_seum_mean* | mmol/L | 110.3 - 170.0 | 19.26% |
|  | annual median value | *lab_Na_seum_median* |  | 104.9 - 170.0 | 19.26% |
|  | annual maximum value | *lab_Na_seum_max* |  | 114.3 - 326.8 | 19.26% |
|  | annual minimum value | *lab_Na_seum_min* |  | 84 - 168 | 19.26% |
|  | availability, binary (yes, no) | *C_lab_Na_seum* |  | 0, 1 | 0 |
| Albumin/Creatinine Ratio (ACR) in Urine | annual average value | *lab_AlbCreaR_mean* | mg/mmol | 0.0 - 12407.1 | 67.18% |
|  | annual median value | *lab_AlbCreaR_median* |  | 0.0 - 12407.1 | 67.18% |
|  | annual maximum value | *lab_AlbCreaR_max* |  | 0.0 - 12407.1 | 67.18% |
|  | annual minimum value | *lab_AlbCreaR_min* |  | 0.0 - 12407.1 | 67.18% |
|  | availability, binary (yes, no) | *C_lab_AlbCreaR* |  | 0, 1 | 0 |
| Alkaline phosphatase (ALP) in Serum or Plasma | annual average value | *lab_ALP_mean* | U/L | 0.0 - 4014.7 | 41.40% |
|  | annual median value | *lab_ALP_median* |  | 0.0 - 3128.0 | 41.40% |
|  | annual maximum value | *lab_ALP_max* |  | 0.0 - 15136.0 | 41.40% |
|  | annual minimum value | *lab_ALP_min* |  | 0.0 - 2242.0 | 41.40% |
|  | availability, binary (yes, no) | *C_lab_ALP* |  | 0, 1 | 0 |
| Hemoglobin A1c (HbA1c) in Blood | annual average value | *lab_HbA1c_all_mean* | % | 3.4 - 20.6 | 54.33% |
|  | annual median value | *lab_HbA1c_all_median* |  | 3.4 - 20.6 | 54.33% |
|  | annual maximum value | *lab_HbA1c_all_max* |  | 3.4 - 20.8 | 54.33% |
|  | annual minimum value | *lab_HbA1c_all_min* |  | 3.4 - 20.6 | 54.33% |
|  | availability, binary (yes, no) | *C_lab_HbA1c_all* |  | 0, 1 | 0 |
| Low-density lipoprotein cholesterol (LDL-C) in Serum or Plasma | annual average value | *lab_LDLC_cal_mean* | mmol/L | 1.8 - 23.7 | 24.72% |
|  | annual median value | *lab_LDLC_cal_median* |  | 1.8 - 23.7 | 24.72% |
|  | annual maximum value | *lab_LDLC_cal_max* |  | 1.9 – 27.0 | 24.72% |
|  | annual minimum value | *lab_LDLC_cal_min* |  | 1.7 - 20.4 | 24.72% |
|  | availability, binary (yes, no) | *C_lab_LDLC_cal* |  | 0, 1 | 0 |
| Globulin in Serum by Calculated | annual average value | *lab_Glob_mean* | g/L | 3.0 - 97.3 | 50.82% |
|  | annual median value | *lab_Glob_median* |  | 3.0 - 98.0 | 50.82% |
|  | annual maximum value | *lab_Glob_max* |  | 3.0 - 124.0 | 50.82% |
|  | annual minimum value | *lab_Glob_min* |  | 1.9 - 92.9 | 50.82% |
|  | availability, binary (yes, no) | *C_lab_Glob* |  | 0, 1 | 0 |
| Estimated glomerular filtration rate (eGFR) calculated by the 2009 Chronic Kidney Disease Epidemiology Collaboration (CKD-EPI) creatinine equation | annual average value | *lab_eGFR_all_mean* | mL/min/1.73m^2^ | 1.80 - 159.90 | 19.07% |
|  | annual median value | *lab_eGFR_all_median* |  | 1.65 - 159.90 | 19.07% |
|  | annual maximum value | *lab_eGFR_all_max* |  | 1.95 - 159.90 | 19.07% |
|  | annual minimum value | *lab_eGFR_all_min* |  | 0.86 - 157.54 | 19.07% |
|  | availability, binary (yes, no) | *C_lab_eGFR_all* |  | 0, 1 | 0 |
| **Medications (dispensed prescription)** | | | British National Formulary (BNF) code |  |  |
| Dyspepsia and gastro-esophageal reflux disease | binary (yes, no) | *drug_1.1* | 1.1 | 0, 1 | 0 |
| Antispasmodics and other drugs altering gut motility | binary (yes, no) | *drug_1.2* | 1.2 | 0, 1 | 0 |
| Antisecretory drugs and mucosal protectants | binary (yes, no) | *drug_1.3* | 1.3 | 0, 1 | 0 |
| Acute diarrhea | binary (yes, no) | *drug_1.4* | 1.4 | 0, 1 | 0 |
| Chronic bowel disorders | binary (yes, no) | *drug_1.5* | 1.5 | 0, 1 | 0 |
| Laxatives | binary (yes, no) | *drug_1.6* | 1.6 | 0, 1 | 0 |
| Local preparations of anal and rectal disorders | binary (yes, no) | *drug_1.7* | 1.7 | 0, 1 | 0 |
| Drugs affecting intestinal secretions | binary (yes, no) | *drug_1.9* | 1.9 | 0, 1 | 0 |
| Positive inotropic drugs | binary (yes, no) | *drug_2.1* | 2.1 | 0, 1 | 0 |
| Diuretics | binary (yes, no) | *drug_2.2* | 2.2 | 0, 1 | 0 |
| Anti-arrhythmic drugs | binary (yes, no) | *drug_2.3* | 2.3 | 0, 1 | 0 |
| Beta-adrenoceptor blocking drugs | binary (yes, no) | *drug_2.4* | 2.4 | 0, 1 | 0 |
| Vasodilator antihypertensive drugs | binary (yes, no) | *drug_2.5.1* | 2.5.1 | 0, 1 | 0 |
| Centrally acting antihypertensive drugs | binary (yes, no) | *drug_2.5.2* | 2.5.2 | 0, 1 | 0 |
| Alpha-adrenoceptor blocking drugs | binary (yes, no) | *drug_2.5.4* | 2.5.4 | 0, 1 | 0 |
| Drugs affecting the renin-angiotensin system | binary (yes, no) | *drug_2.5.5* | 2.5.5 | 0, 1 | 0 |
| Nitrates, calcium-channel blockers, and other antianginal drugs | binary (yes, no) | *drug_2.6* | 2.6 | 0, 1 | 0 |
| Sympathomimetics | binary (yes, no) | *drug_2.7* | 2.7 | 0, 1 | 0 |
| Anticoagulants and protamine | binary (yes, no) | *drug_2.8* | 2.8 | 0, 1 | 0 |
| Antiplatelet drugs | binary (yes, no) | *drug_2.9* | 2.9 | 0, 1 | 0 |
| Antifibrinolytic drugs and hemostatic | binary (yes, no) | *drug_2.11* | 2.11 | 0, 1 | 0 |
| Lipid-regulating drugs | binary (yes, no) | *drug_2.12* | 2.12 | 0, 1 | 0 |
| Miscellaneous (cardiovascular system) | binary (yes, no) | *drug_2.A* | 2.A | 0, 1 | 0 |
| Bronchodilators | binary (yes, no) | *drug_3.1* | 3.1 | 0, 1 | 0 |
| Corticosteroids | binary (yes, no) | *drug_3.2* | 3.2 | 0, 1 | 0 |
| Cromoglicate, leukotriene receptor antagonist & phosphodiesterase type-4 inhibitor | binary (yes, no) | *drug_3.3* | 3.3 | 0, 1 | 0 |
| Antihistamines, hypersensitization, and allergic emergencies | binary (yes, no) | *drug_3.4* | 3.4 | 0, 1 | 0 |
| Mucolytics | binary (yes, no) | *drug_3.7* | 3.7 | 0, 1 | 0 |
| Cough preparations | binary (yes, no) | *drug_3.9* | 3.9 | 0, 1 | 0 |
| Miscellaneous (respiratory system) | binary (yes, no) | *drug_3.A* | 3.A | 0, 1 | 0 |
| Hypnotics and anxiolytics | binary (yes, no) | *drug_4.1* | 4.1 | 0, 1 | 0 |
| Drugs for dementia | binary (yes, no) | *drug_4.11* | 4.11 | 0, 1 | 0 |
| Drugs used in psychoses and related disorders | binary (yes, no) | *drug_4.2* | 4.2 | 0, 1 | 0 |
| Antidepressant drugs | binary (yes, no) | *drug_4.3* | 4.3 | 0, 1 | 0 |
| Drugs used in nausea and vertigo | binary (yes, no) | *drug_4.6* | 4.6 | 0, 1 | 0 |
| Analgesics | binary (yes, no) | *drug_4.7* | 4.7 | 0, 1 | 0 |
| Antiepileptic drugs | binary (yes, no) | *drug_4.8* | 4.8 | 0, 1 | 0 |
| Drugs used in parkinsonism and related disorders | binary (yes, no) | *drug_4.9* | 4.9 | 0, 1 | 0 |
| Antibacterial drugs | binary (yes, no) | *drug_5.1* | 5.1 | 0, 1 | 0 |
| Antifungal drugs | binary (yes, no) | *drug_5.2* | 5.2 | 0, 1 | 0 |
| Antiviral drugs | binary (yes, no) | *drug_5.3* | 5.3 | 0, 1 | 0 |
| Insulins | binary (yes, no) | *drug_6.1.1* | 6.1.1 | 0, 1 | 0 |
| Sulfonylureas | binary (yes, no) | *drug_6.1.2.1* | 6.1.2.1 | 0, 1 | 0 |
| Biguanides (metformin) | binary (yes, no) | *drug_6.1.2.2* | 6.1.2.2 | 0, 1 | 0 |
| Alpha-glucosidase inhibitors (AGI) | binary (yes, no) | *drug_AGI* | 6.1.2.3.1 | 0, 1 | 0 |
| Combinations of oral anti-diabetic drugs (OADs) | binary (yes, no) | *drug_Com* | 6.1.2.3.2 | 0, 1 | 0 |
| Dipeptidyl peptidase-4 inhibitors (DPP4-i) | binary (yes, no) | *drug_DPP4* | 6.1.2.3.3 | 0, 1 | 0 |
| Glucagon-like peptide-1 receptor agonists (GLP1-RA) | binary (yes, no) | *drug_GLP1* | 6.1.2.3.4 | 0, 1 | 0 |
| Sodium-glucose cotransporter 2 inhibitors (SGLT2-i) | binary (yes, no) | *drug_SGLT2* | 6.1.2.3.5 | 0, 1 | 0 |
| Thiazolidinediones (TZD) | binary (yes, no) | *drug_TZD* | 6.1.2.3.6 | 0, 1 | 0 |
| Thyroid and antithyroid drugs | binary (yes, no) | *drug_6.2* | 6.2 | 0, 1 | 0 |
| Corticosteroids | binary (yes, no) | *drug_6.3* | 6.3 | 0, 1 | 0 |
| Sex hormones | binary (yes, no) | *drug_6.4* | 6.4 | 0, 1 | 0 |
| Hypothalamic and pituitary hormones and anti-estrogens | binary (yes, no) | *drug_6.5* | 6.5 | 0, 1 | 0 |
| Drugs affecting bone metabolism | binary (yes, no) | *drug_6.6* | 6.6 | 0, 1 | 0 |
| Other endocrine drugs | binary (yes, no) | *drug_6.7* | 6.7 | 0, 1 | 0 |
| Treatment of vaginal and vulval conditions | binary (yes, no) | *drug_7.2* | 7.2 | 0, 1 | 0 |
| Drugs for genito-urinary disorders | binary (yes, no) | *drug_7.4* | 7.4 | 0, 1 | 0 |
| Cytotoxic drugs | binary (yes, no) | *drug_8.1* | 8.1 | 0, 1 | 0 |
| Drugs affecting the immune response | binary (yes, no) | *drug_8.2* | 8.2 | 0, 1 | 0 |
| Sex hormones and hormone antagonists in malignant disease | binary (yes, no) | *drug_8.3* | 8.3 | 0, 1 | 0 |
| Anemias and some other blood disorders | binary (yes, no) | *drug_9.1* | 9.1 | 0, 1 | 0 |
| Fluids and electrolytes | binary (yes, no) | *drug_9.2* | 9.2 | 0, 1 | 0 |
| Intravenous nutrition | binary (yes, no) | *drug_9.3* | 9.3 | 0, 1 | 0 |
| Oral nutrition | binary (yes, no) | *drug_9.4* | 9.4 | 0, 1 | 0 |
| Minerals | binary (yes, no) | *drug_9.5* | 9.5 | 0, 1 | 0 |
| Vitamins | binary (yes, no) | *drug_9.6* | 9.6 | 0, 1 | 0 |
| Miscellaneous (nutrition and blood) | binary (yes, no) | *drug_9.A* | 9.A | 0, 1 | 0 |
| Drugs used in rheumatic diseases and gout | binary (yes, no) | *drug_10.1* | 10.1 | 0, 1 | 0 |
| Drugs used in neuromuscular disorders | binary (yes, no) | *drug_10.2* | 10.2 | 0, 1 | 0 |
| Drugs for the treatment of soft-tissue disorders and topical pain relief | binary (yes, no) | *drug_10.3* | 10.3 | 0, 1 | 0 |
| Anti-infective eye preparations | binary (yes, no) | *drug_11.3* | 11.3 | 0, 1 | 0 |
| Corticosteroids and other anti-inflammatory preparations | binary (yes, no) | *drug_11.4* | 11.4 | 0, 1 | 0 |
| Mydriatics and cycloplegics | binary (yes, no) | *drug_11.5* | 11.5 | 0, 1 | 0 |
| Treatment of glaucoma | binary (yes, no) | *drug_11.6* | 11.6 | 0, 1 | 0 |
| Local anesthetics | binary (yes, no) | *drug_11.7* | 11.7 | 0, 1 | 0 |
| Miscellaneous ophthalmic preparations | binary (yes, no) | *drug_11.8* | 11.8 | 0, 1 | 0 |
| Miscellaneous (eye) | binary (yes, no) | *drug_11.A* | 11.A | 0, 1 | 0 |
| Drugs acting on the ear | binary (yes, no) | *drug_12.1* | 12.1 | 0, 1 | 0 |
| Drugs acting on the nose | binary (yes, no) | *drug_12.2* | 12.2 | 0, 1 | 0 |
| Drugs acting on the oropharynx | binary (yes, no) | *drug_12.3* | 12.3 | 0, 1 | 0 |
| Miscellaneous (ear, nose, and oropharynx) | binary (yes, no) | *drug_12.A* | 12.A | 0, 1 | 0 |
| Skin | binary (yes, no) | *drug_13* | 13 | 0, 1 | 0 |
| Immunological Products and Vaccines | binary (yes, no) | *drug_14* | 14 | 0, 1 | 0 |
| Anesthesia | binary (yes, no) | *drug_15* | 15 | 0, 1 | 0 |
| Disinfectants / Cleansing Agents | binary (yes, no) | *drug_C* | C | 0, 1 | 0 |
| Miscellaneous Drugs | binary (yes, no) | *drug_E* | E | 0, 1 | 0 |

**Note 1*** Hospital Authority-wide standardized specialty ID: 1=Medicine, 2=Clinical oncology, 3=Surgery, 4=Orthopedics & trauma, 5=Ear, nose and throat, 6=Ophthalmology, 7=Neurosurgery, 8=Cardiothoracic Surgery, 9=Gynecology, 10=GOP, 11=Obstetrics, 12=Pediatrics and Adolescent Medicine, 13=Psychiatry, 14=Audiology, 15=Staff Clinic, 16=Family medicine, 17=Optometry, 18=Orthoptics, 19=Multi_AH Departments, 20=Dental, 21=Community Medicine, 22=Multi-Specialty, 23=Pathology, 24=A&E, 25=Clinical Psychology, 26=Rehabilitation, 27=Hospice, 28=Other/Unclassified, 29=Podiatry, 30=Dietetics, 31=Medical Social Work, 32=Occupational Therapy, 33=Pharmacy, 34=Physiotherapy, 35=Prosthetics & Orthotics, 36=Speech Therapy, 37=Unclassified (Division).
