# Supplementary material for: A novel electronic health record-based, machine-learning model to predict severe hypoglycemia leading to hospitalizations in older adults with diabetes: A territory-wide cohort and modeling study
Source: PLoS Med. 2024 Apr 12;21(4):e1004369. doi: 10.1371/journal.pmed.1004369 (PMC11014435; doi:10.1371/journal.pmed.1004369)
Supplement: S3 Table — (DOCX) [file pmed.1004369.s005.docx]

### S3 Table. Hyper-parameter values for machine-learning (ML) models.

| **ML algorithm** | **Hyper-parameter** | **Range of values for grid search** | **Optimal values for the 258-variable model** |
| --- | --- | --- | --- |
| GLM | *alpha* | 0 – 1, step 0.01 | 0.09 |
|  | *lambda* | 0 – 1, step 0.01 | 0.0012 |
| DRF | *sample_rate* | 0.5 – 1.0, step 0.01 | 0.5 |
|  | *col_sample_rate_per_tree* | 0.8 – 1.0, step 0.01 | 0.8 |
|  | *mtries* | 10 – 20, step 1 | 16 |
|  | *min_rows* | 1, 2, 3 | 3 |
|  | *max_depth* | 10 – 30, step 1 | 14 |
|  | *ntrees* | 50 – 200, step 1 | 34 |
| GBM | *learn_rate* | 0.05 – 0.2, step 0.01 | 0.15 |
|  | *max_depth* | 3, 4, 5, 6 | 4 |
|  | *sample_rate* | 0.7 – 1.0, step 0.01 | 0.87 |
|  | *col_sample_rate* | 0.8 – 1.0, step 0.01 | 1 |
|  | *ntrees* | 50 – 200, step 1 | 160 |
| Rulefit* | *Default* |  |  |
| DNN | *epochs* | 5 – 20, step 1 | 14 |
|  | *hidden* | 100, 150, 200, 250 | 100 |
|  | *target_ratio_comm_to_comp* | 0.05, 0.25, 0.5 | 0.25 |
|  | *l1* | 0 – 1, step 0.01 | 0.99 |
|  | *l2* | 0 – 1, step 0.01 | 0.72 |
|  | *hidden_dropout_ratios* | 0 – 1, step 0.01 | 0.09 |
|  | *input_dropout_ratio* | 0 – 0.3, step 0.01 | 0.0 |
|  | *rho* | 0.5 – 1, step 0.01 | 0.65 |
| XGBoost | *ntrees* | 20 – 200, step 1 | 167 |
|  | *max_depth* | 3 – 10, step 1 | 5 |
|  | *min_rows* | 1 – 4, step 1 | 4 |
|  | *learn_rate* | 0 – 1, step 0.01 | 0.25 |
|  | *sample_rate* | 0 – 1, step 0.01 | 0.8 |
|  | *col_sample_rate* | 0 – 1, step 0.01 | 0.9 |
|  | *col_sample_rate_per_tree* | 0 – 1, step 0.01 | 0.9 |
|  | *colsample_bynode* | 0 – 1, step 0.01 | 0.8 |

GLM, generalized linear model; DRF, distributed random forest; GBM, gradient boosting machine; DNN, deep neural network; XGBoost, extreme gradient boosting. The optimal values that yielded the best AUPRC in testing data for each class of ML algorithm were recorded. *: grid search was not available for the Rulefit algorithm in H2O; default setting was thus used.
