# Supplementary material for: A novel electronic health record-based, machine-learning model to predict severe hypoglycemia leading to hospitalizations in older adults with diabetes: A territory-wide cohort and modeling study
Source: PLoS Med. 2024 Apr 12;21(4):e1004369. doi: 10.1371/journal.pmed.1004369 (PMC11014435; doi:10.1371/journal.pmed.1004369)
Supplement: S4 Table — (DOCX) [file pmed.1004369.s006.docx]

### S4 Table. Cohort characteristics by outcome and dataset.

| **Characteristics** | **By one-year SH hospitalization event** | | |  | **By dataset** | | | | |
| --- | --- | --- | --- | --- | --- | --- | --- | --- | --- |
|  | **with**  **event** | **without**  **event** | ***P* value** |  | **training** | **testing** | **validation** | ***P* value** | **temporal validation** |
| Number of patients | 11,128 | 1,446,223 | <0.001 |  | 1,020,051 | 291,569 | 145,731 | 1.000 | 13,917 |
| Outcome events in 12 months (%) | 11,128 (100) | 0 |  |  | 7,792 (0.7) | 2,225 (0.7) | 1,111 (0.7) | 0.998 | 722 (5.2) |
| Year (%) |  |  | <0.001 |  |  |  |  | <0.001 |  |
| 2013 | 3,467 (31.2) | 265,350 (18.3) |  |  | 188,155 (18.4) | 53,781 (18.4) | 26,881 (18.4) |  | 0 |
| 2014 | 2,495 (22.4) | 282,062 (19.5) |  |  | 199,171 (19.5) | 56,931 (19.5) | 28,455 (19.5) |  | 0 |
| 2015 | 1,981 (17.8) | 292,396 (20.2) |  |  | 206,045 (20.2) | 58,895 (20.2) | 29,437 (20.2) |  | 0 |
| 2016 | 1,704 (15.3) | 300,659 (20.8) |  |  | 211,634 (20.7) | 60,494 (20.7) | 30,235 (20.7) |  | 0 |
| 2017 | 1,481 (13.3) | 305,756 (21.1) |  |  | 215,046 (21.1) | 61,468 (21.1) | 30,723 (21.1) |  | 0 |
| Male (%) | 4,884 (43.9) | 673,773 (46.6) | <0.001 |  | 474,472 (46.5) | 135,818 (46.6) | 68,015 (46.7) | 0.622 | 7,183 (50.2) |
| Age; mean (SD) | 77.9 (7.6) | 74.4 (8.0) | <0.001 |  | 74.4 (8.0) | 74.4 (8.0) | 74.4 (8.0) | 0.520 | 73.6 (7.4) |
| Age group (%) |  |  | <0.001 |  |  |  |  | 0.076 |  |
| 65-69 | 1,932 (17.4) | 493,918 (34.2) |  |  | 347,014 (34.0) | 99,089 (34.0) | 49,687 (34.1) |  | 5,264 (36.8) |
| 70-79 | 4,184 (37.6) | 546,812 (37.8) |  |  | 384,979 (37.8) | 110,439 (37.9) | 55,301 (38.0) |  | 5,792 (40.5) |
| 80-89 | 4,434 (39.9) | 351,583 (24.3) |  |  | 249,228 (24.4) | 71,120 (24.4) | 35,321 (24.2) |  | 2,839 (19.9) |
| 90+ | 576 (5.2) | 53,910 (3.7) |  |  | 38,317 (3.8) | 10,774 (3.7) | 5,347 (3.7) |  | 400 (2.8) |
| In-patient records; mean (SD) | 3.7 (13.9) | 2.1 (9.1) | <0.001 |  | 2.1 (9.1) | 2.2 (9.3) | 2.1 (9.7) | 0.724 | 1.1 (5.4) |
| Out-patient records; mean (SD) | 18.2 (20.9) | 15.1 (16.1) | <0.001 |  | 15.1 (16.2) | 15.2 (16.0) | 15.1 (15.7) | 0.146 | 9.8 (11.1) |
| Glucose-lowering drugs (%) |  |  |  |  |  |  |  |  |  |
| Metformin | 6,860 (61.6) | 938,895 (64.9) | <0.001 |  | 661,267 (64.9) | 189,082 (64.9) | 95,056 (65.3) | 0.818 | 8,762 (61.3) |
| Sulfonylurea | 7,500 (67.4) | 627,207 (43.4) | <0.001 |  | 443,960 (43.5) | 126,637 (43.5) | 63,744 (43.8) | 0.832 | 6,443 (45.1) |
| DPP4-i | 1,885 (16.9) | 112,400 (7.8) | <0.001 |  | 79,700 (7.8) | 22,807 (7.8) | 11,561 (7.9) | 0.388 | 2,487 (17.4) |
| TZD | 248 (2.2) | 14,801 (1.0) | <0.001 |  | 10,421 (1.0) | 3,043 (1.0) | 1,547 (1.1) | 0.971 | 718 (5.0) |
| GLP1-RA | 6 (0.1) | 437 (<0.1) | 0.248 |  | 320 (<0.1) | 75 (<0.1) | 47 (<0.1) | 0.444 | 99 (0.7) |
| SGLT2-i | 29 (0.3) | 4,518 (0.3) | 0.373 |  | 3,202 (0.3) | 885 (0.3) | 454 (0.3) | 0.345 | 949 (6.6) |
| Insulin | 5,369 (48.2) | 177,700 (12.3) | <0.001 |  | 127,773 (12.5) | 36,401 (12.5) | 18,188 (12.5) | 0.354 | 3,968 (27.8) |
| Lipid-regulating drugs | 1,658 (14.9) | 978,625 (67.7) | <0.001 |  | 196,670 (67.5) | 688,636 (67.5) | 98,693 (67.8) | 0.241 | 10,034 (72.1) |
| History of insulin (%) | 5,571 (50.1) | 188,358 (13.0) | <0.001 |  | 135,340 (13.3) | 38,560 (13.2) | 19,265 (13.2) | 0.459 | 5,151 (36.0) |
| History of SH (%) | 1,116 (10.0) | 10,382 (0.7) | <0.001 |  | 7,661 (0.8) | 2,100 (0.7) | 1,070 (0.7) | 0.218 | 509 (3.6) |

Abbreviations: SH, severe hypoglycemia; SD, standard deviation; IQR, interquartile range; DPP4-i, dipeptidyl peptidase-4 inhibitors; TZD, thiazolidinediones; GLP1-RA, glucagon-like peptide-1 receptor agonists; SGLT2-i, sodium-glucose cotransporter 2 inhibitors.
