# Supplementary material for: A novel electronic health record-based, machine-learning model to predict severe hypoglycemia leading to hospitalizations in older adults with diabetes: A territory-wide cohort and modeling study
Source: PLoS Med. 2024 Apr 12;21(4):e1004369. doi: 10.1371/journal.pmed.1004369 (PMC11014435; doi:10.1371/journal.pmed.1004369)
Supplement: S5 Table — (DOCX) [file pmed.1004369.s007.docx]

### S5 Table. Performance metrics of sensitivity analyses and 11-variable models.

| **Machine learning models** | **Model** | **AUROC** | **AUPRC** | **PPV** | **NPV** | **F1** | **MCC** | |  |
| --- | --- | --- | --- | --- | --- | --- | --- | --- | --- |
| **Sensitivity Analysis I: Top 30 predictors** | | | | | | | | | |
| Training set | XGBoost | 0.982 | 0.704 | 0.833 | 0.997 | 0.671 | 0.682 | |  |
| Testing set |  | 0.975 | 0.655 | 0.785 | 0.996 | 0.636 | 0.645 | |  |
| **Validation set** |  | **0.975** | **0.632** | **0.763** | **0.996** | **0.610** | **0.620** | |  |
| Temporal validation set |  | 0.875 | 0.260 | 0.263 | 0.973 | 0.354 | 0.329 | |  |
| **Sensitivity Analysis II: 22 predictors selected from the top 30 predictors** | | | | | | | |  |  |
| Training set | XGBoost | 0.969 | 0.615 | 0.746 | 0.996 | 0.604 | 0.613 | |  |
| Testing set |  | 0.960 | 0.553 | 0.796 | 0.996 | 0.557 | 0.582 | |  |
| **Validation set** |  | **0.959** | **0.540** | **0.765** | **0.996** | **0.544** | **0.566** | |  |
| Temporal validation set |  | 0.788 | 0.158 | 0.134 | 0.974 | 0.221 | 0.209 | |  |
| **Sensitivity Analysis III: 1^st^ occurrence of outcome event in each independent prediction horizon** | | | | | | | |  |  |
| Training set | XGBoost | 0.983 | 0.702 | 0.766 | 0.997 | 0.663 | 0.668 | |  |
| Testing set |  | 0.971 | 0.610 | 0.738 | 0.996 | 0.595 | 0.605 | |  |
| **Validation set** |  | **0.972** | **0.636** | **0.799** | **0.996** | **0.628** | **0.641** | |  |
| **11-variable models** | | | | | | | |  |  |
| Validation set | XGBoost (default) | 0.938 | 0.280 |  |  |  |  | |  |
|  | DRF (tuned) | 0.936 | 0.276 |  |  |  |  | |  |
|  | RuleFit (default) | 0.936 | 0.273 |  |  |  |  | |  |
|  | XGBoost (tuned) | 0.934 | 0.269 |  |  |  |  | |  |
|  | GBM (tuned) | 0.935 | 0.266 |  |  |  |  | |  |
|  | GBM (default) | 0.938 | 0.265 |  |  |  |  | |  |
|  | DRF (default) | 0.929 | 0.263 |  |  |  |  | |  |
|  | DNN (default) | 0.908 | 0.150 |  |  |  |  | |  |
|  | GLM (default) | 0.907 | 0.094 |  |  |  |  | |  |
|  | GLM (tuned) | 0.907 | 0.088 |  |  |  |  | |  |
|  | DNN (tuned) | 0.845 | 0.070 |  |  |  |  | |  |

Abbreviations: AUROC, area under the receiver operating characteristic curve; AUPRC, area under the precision-recall curve; PPV, positive predictive value (i.e., precision); NPV, negative predictive value; F1, F1 score calculated from the harmonic mean of the precision and recall; MCC, Matthews Correlation Coefficient. GLM, generalized linear model; DRF, distributed random forest; GBM, gradient boosting machine; DNN, deep neural network; XGBoost, extreme gradient boosting. Settings of hyper-parameter are provided in parentheses.
