# Supplementary material for: A novel electronic health record-based, machine-learning model to predict severe hypoglycemia leading to hospitalizations in older adults with diabetes: A territory-wide cohort and modeling study
Source: PLoS Med. 2024 Apr 12;21(4):e1004369. doi: 10.1371/journal.pmed.1004369 (PMC11014435; doi:10.1371/journal.pmed.1004369)
Supplement: S6 Table — (DOCX) [file pmed.1004369.s008.docx]

### S6 Table. Scaled score for risk stratification in the validation set.

| Scaled score | Percentage of outcome events below the score (%) |
| --- | --- |
| 1 | 0.00 |
| 2 | 0.00 |
| 3 | 0.00 |
| 4 | 0.00 |
| 5 | 0.00 |
| 6 | 0.00 |
| 7 | 0.00 |
| 8 | 0.00 |
| 9 | 0.00 |
| 10 | 0.00 |
| 11 | 0.00 |
| 12 | 0.00 |
| 13 | 0.10 |
| 14 | 0.10 |
| 15 | 0.10 |
| 16 | 0.10 |
| 17 | 0.19 |
| 18 | 0.19 |
| 19 | 0.19 |
| 20 | 0.19 |
| 21 | 0.19 |
| 22 | 0.19 |
| 23 | 0.19 |
| 24 | 0.19 |
| 25 | 0.19 |
| 26 | 0.19 |
| 27 | 0.19 |
| 28 | 0.19 |
| 29 | 0.29 |
| 30 | 0.29 |
| 31 | 0.29 |
| 32 | 0.39 |
| 33 | 0.48 |
| 34 | 0.58 |
| 35 | 0.58 |
| 36 | 0.58 |
| 37 | 0.58 |
| 38 | 0.58 |
| 39 | 0.58 |
| 40 | 0.58 |
| 41 | 0.58 |
| 42 | 0.58 |
| 43 | 0.58 |
| 44 | 0.58 |
| 45 | 0.68 |
| 46 | 0.77 |
| 47 | 0.77 |
| 48 | 0.87 |
| 49 | 0.97 |
| 50 | 0.97 |
| 51 | 1.06 |
| 52 | 1.16 |
| 53 | 1.16 |
| 54 | 1.35 |
| 55 | 1.45 |
| 56 | 1.54 |
| 57 | 1.54 |
| 58 | 1.64 |
| 59 | 1.74 |
| 60 | 1.93 |
| 61 | 2.03 |
| 62 | 2.03 |
| 63 | 2.22 |
| 64 | 2.32 |
| 65 | 2.41 |
| 66 | 2.41 |
| 67 | 2.70 |
| 68 | 2.99 |
| 69 | 3.28 |
| 70 | 3.67 |
| 71 | 3.76 |
| 72 | 3.76 |
| 73 | 3.86 |
| 74 | 3.86 |
| 75 | 3.96 |
| 76 | 4.05 |
| 77 | 4.44 |
| 78 | 4.83 |
| 79 | 5.41 |
| 80 | 5.89 |
| 81 | 6.27 |
| 82 | 6.76 |
| 83 | 7.24 |
| 84 | 8.30 |
| 85 | 9.56 |
| **86** | **10.04** |
| 87 | 11.10 |
| 88 | 11.58 |
| 89 | 12.55 |
| 90 | 13.22 |
| 91 | 14.67 |
| 92 | 15.83 |
| 93 | 16.89 |
| 94 | 19.40 |
| 95 | 21.33 |
| 96 | 23.94 |
| 97 | 27.70 |
| 98 | 32.24 |
| 99 | 41.02 |
| 100 | 100.00 |
